# The left side of motor resonance

**DOI:** 10.3389/fnhum.2014.00702

**Published:** 2014-09-09

**Authors:** Luisa Sartori, Chiara Begliomini, Giulia Panozzo, Alice Garolla, Umberto Castiello

**Affiliations:** ^1^Dipartimento di Psicologia Generale, Università degli Studi di PadovaPadova, Italy; ^2^Cognitive Neuroscience Center, Università degli Studi di PadovaPadova, Italy

**Keywords:** action observation, motor resonance, complementary actions, handedness, transcranial magnetic stimulation, motor evoked potentials

## Abstract

Motor resonance is defined as the internal activation of an observer's motor system, specifically attuned to the perceived movement. In social contexts, however, different patterns of observed and executed muscular activation are frequently required. This is the case, for instance, of seeing a key offered with a precision grip and received by opening the hand. Novel evidence suggests that compatibility effects in motor resonance can be altered by social response preparation. What is not known is how handedness modulates this effect. The present study aimed at determining how a left- and a right-handed actor grasping an object and then asking for a complementary response influences corticospinal activation in left- and right-handers instructed to observe the scene. Transcranial magnetic stimulation (TMS)-induced motor evoked potentials (MEPs) were thus recorded from the dominant hands of left- and right-handers. Interestingly, requests posed by the right-handed actor induced a motor activation in the participants' respective dominant hands, suggesting that left-handers tend to mirror right-handers with their most efficient hand. Whereas requests posed by the left-handed actor activated the anatomically corresponding muscles (i.e., left hand) in all the participants, right-handers included. Motor resonance effects classically reported in the literature were confirmed when observing simple grasping actions performed by the right-handed actor. These findings indicate that handedness influences both congruent motor resonance and complementary motor preparation to observed actions.

## Introduction

A considerable amount of data suggests that primary motor and somatosensory cortices, as well as premotor and parietal areas, are modulated during action observation, providing evidence of an activation of the observer's motor system (i.e., motor resonance; see for example Grèzes and Decety, 2001; Avenanti et al., 2007, 2013a,b). Motor resonance is thought to result from the activity of neurons homologous to the mirror neurons described in the monkey ventral premotor cortex (di Pellegrino et al., 1992; Gallese et al., 1996). In humans, a large number of functional magnetic resonance imaging (fMRI) studies have provided reliable evidence that the action observation network (i.e., the neural network activated by seeing others' actions) largely overlaps with the brain network involved in action execution (Etzel et al., 2008; Gazzola and Keysers, 2009; Kilner et al., 2009; Turella et al., 2009; Oosterhof et al., 2010). Moreover, transcranial magnetic-stimulation (TMS) studies have shown a corticospinal excitability facilitation during action observation, suggesting a role for the primary motor area (M1) in motor resonance (Fadiga et al., 1995; Strafella and Paus, 2000; Gangitano et al., 2001; Aziz-Zadeh et al., 2002; Clark et al., 2004; Catmur et al., 2007; Enticott et al., 2010, 2011; Senot et al., 2011). In neural terms, the resonant response would originate in inferior frontal cortex (IFC, including ventral premotor cortex and posterior part of inferior frontal gyrus) and in inferior parietal lobule (IPL), and descend to spinal motoneurones via M1 (Nishitani and Hari, 2000). This is demonstrated by perturb-and-measure studies (Paus, 2005; Avenanti et al., 2007) in which off-line suppression of neural activity in IFC disrupts the motor facilitation induced by action observation (Avenanti et al., 2007, 2013a,b; Enticott et al., 2012) and dual coil studies in which stimulation of IFC and IPL modulates motor cortex reactivity to observed actions (Koch et al., 2010; Catmur et al., 2011). The involvement of M1 has been further confirmed by experiments in which the left M1 hand area was temporarily inactivated by TMS conditioning, resulting in the loss of the resonant H-reflex modulation in the corresponding right hand muscle (Borroni and Baldissera, 2008). Much of this work involved magnetic stimulation of the human primary motor cortex (M1) and electromyography (EMG) recording of participants' contralateral hand muscles while they were watching hand movements. The amplitude of motor evoked potentials (MEPs) recorded from hand muscles was found to be increased during observation of others' actions as the product of a specific corticospinal (CS) facilitation. In this connection, a question which so far has received little attention is whether the tendency to automatically resonate with others' actions is inflexible in terms of handedness. To date, as left-handed participants have often been excluded from studies in the past, our understanding of the relationship between motor resonance and motor dominance is quite limited. Preliminary evidence paved the way indicating that observation of a hand movement can modulate the excitability of motor neurons innervating hand muscles of both sides, irrespective of whether the right or left hand is observed (Borroni et al., 2008). Such a bilateral involvement indicates that motor resonance is not limited to a one-to-one correspondence, but it evokes the subliminal implementation of the full activation pattern utilized during execution, including other limbs' muscles. In this light, it is possible that the premotor cortex is engaged bilaterally in motor resonance during observation of either left or right hands because it does not code the laterality of the observed hand, but a more abstract representation of the movement (Borroni et al., 2008). On the other hand, brain imaging studies have reported the importance of the *observers*' hand dominance in shaping the pattern of motor resonant responses (e.g., Cabinio et al., 2010). In particular, right-handers showed a left-lateralized activation of the mirror neuron system (MNS) when observing/performing a right hand grasp, and a more bilateral but still left-lateralized cortical pattern when observing/performing the same action with the left (non-dominant) hand. The opposite pattern of cortical activation was shown in left-handers, although less lateralized. Along these lines, a series of fMRI studies assessed the role of handedness during execution and observation of simple movements in right- and left-handed participants (Rocca et al., 2008; Rocca and Filippi, 2010). Results showed different pattern of activations of the MNS in left-handers during the performance of movements with their dominant upper and lower limbs, suggesting a complex interaction between innate and daily-life background. These findings support the notion that left-handers can adapt their actions to a world that has been built for right-handed people and that they deal with the vast majority of common tools by simply mirroring right-handers (Rocca et al., 2008).

Support to this contention comes from a recent study in which TMS-induced MEPs were recorded from the dominant and non-dominant hands of left-and right-handed participants while they observed a left-or a right-handed actor grasping an object (Sartori et al., 2013a). The anatomical correspondence between the observed and the observer's effector classically reported in the literature on motor resonance was confirmed in the dominant hand of both left-as well as right-handers observing actors with their same hand preference. But when the observed and observers' hand preference was mismatched, that anatomical correspondence disappeared. In particular, motor resonance was noted in left handers' dominant effector while they were observing both right- and left-handed actors. This seems to suggest a propensity to functionally shift the motor resonant activation to their own dominant hand, in line with neural evidence of more bilaterally spread brain functions in left-than in right-handers (Matsuo et al., 2002; Jorgens et al., 2007; Krombholz, 2008; Müller et al., 2011). The observer's handedness shapes the motor resonant response. What is still unknown, then, is whether the same mechanism applies when a different rather than a similar action is elicited by the observed agent. That is, when an actor is shown leaning toward the observer in a request gesture implying a complementary response.

In specific social contexts requiring incongruent complementary rather than imitative forms of interaction, motor resonance to action observation can be an unsuitable response (for reviews, see Sebanz et al., 2006; Knoblich et al., 2011). For instance, when we observe someone handing us a mug holding it by its handle, we will, without thinking, grab the mug with a whole-hand-grasp (the most appropriate gesture to perform in this situation, though different from that observed). Along these lines, recent evidence seems to suggest that the inflexible tendency to match observed actions onto our motor system can be reconciled with the request to prepare incongruent responses (Newman-Norlund et al., 2007; Ocampo et al., 2011; Hamilton, 2013). In a series of recent psychophysiological studies, researchers assessed CS facilitation while participants observed video-clips evoking complementary gestures (i.e., an actor pouring coffee/sugar and then inviting them to pick up a cup placed in the video foreground) and video-clips simply showing an actor pouring coffee/sugar and then coming back to the starting position (Sartori et al., 2012, 2013b,c,d). Consistent results showed a natural switch from an imitative to a context-related action in CS activity. A matching mechanism at the beginning of an action sequence turned into a complementary one if a request to the observer for a reciprocal action became evident. In particular, TMS-induced MEPs recorded at the time the observer initially perceived a grasp on a target object elicited a motor facilitation in the participant's corresponding hand muscles. Conversely, when the observed gesture elicited a complementary reaction in the observer, participants' hand muscles revealed an activation matching the socially appropriate response which could be performed. As expected, when the observed action did not convey any request to the observer, congruent facilitation effects emerged during action observation.

Capitalizing on these results and recent insights from the handedness literature (Borroni et al., 2008; Rocca et al., 2008; Sartori et al., 2013a), the present study was designed to specifically determine how CS facilitation is modulated when an individual with the same or a different hand dominance elicits a congruent or incongruent motor resonance in the observer. TMS-induced MEPs were then recorded from muscles of each hand per block as the participants watched video-clips. Because participants remained at rest throughout the task, the degree to which the motor system is activated provides an index of CS activity elicited by action observation. Half of the clips showed an actor reaching and grasping an object with her right hand, pouring something and then either coming back (non-social action) or leaning toward an out-of-reach cup crucially located close to the observer and then prompting a complementary response (social action); the other half displayed the same actor performing the same action with her left hand. We expect that observing an actor with a different hand preference might elicit different patterns of CS activation in right- and left-handers. Specifically, if left-handers are prone to functionally shift the motor resonant and complementary activation to their own dominant hand, then leftward activations should be noticed in all the experimental conditions. Otherwise, if handedness does not shape motor resonance, a mirroring pattern of CS facilitation should be found in all the participants. To date, no previous studies have investigated handedness and motor resonance in social contexts by means of TMS and EMG recording. In terms of action observation this might be a timely and tractable issue.

## Materials and methods

### Participants

Thirty right-handed (17 females and 13 males, mean age 24 years, range 19–56) and 30 left-handed (24 females and 6 males, mean age 23 years, range 20–47) participants took part in the experiment. The participants' degree of handedness was evaluated using a modified version of the Edinburgh Inventory (EHI) (Oldfield, 1971; Salmaso and Longoni, 1983). We converted the EHI total score into a dichotomous variable by computing the laterality quotient (LQ) that ranges from −100 (strong left handedness) to +100 (strong right-handedness), through the following standard expression: LQ = (R−L)/(R+L)*100. R and L represent the total number of right-and left-hand items endorsed, respectively. A score below 0 (included) identified left-handed participants, while LQ > 0 detected right-handed participants. The LQ ranged between −100 and −11 (mean: −65) for the left-handed participants. For the right-handed participants, it ranged between 64 and 100 (mean: 88). None of the participants had any neurological, psychiatric, or other medical problems, nor did they have any contraindication to TMS (Wassermann, 1998; Rossi et al., 2009). None were aware of the experiment's purpose and all gave their written informed consent at the time they were recruited. The study protocol was approved by the Ethics Committee of the University of Padova and was carried out in accordance with the principles of the Declaration of Helsinki. None of the participants reported experiencing discomfort or adverse effects during the experiment.

### Experimental stimuli

**Figure 1:**
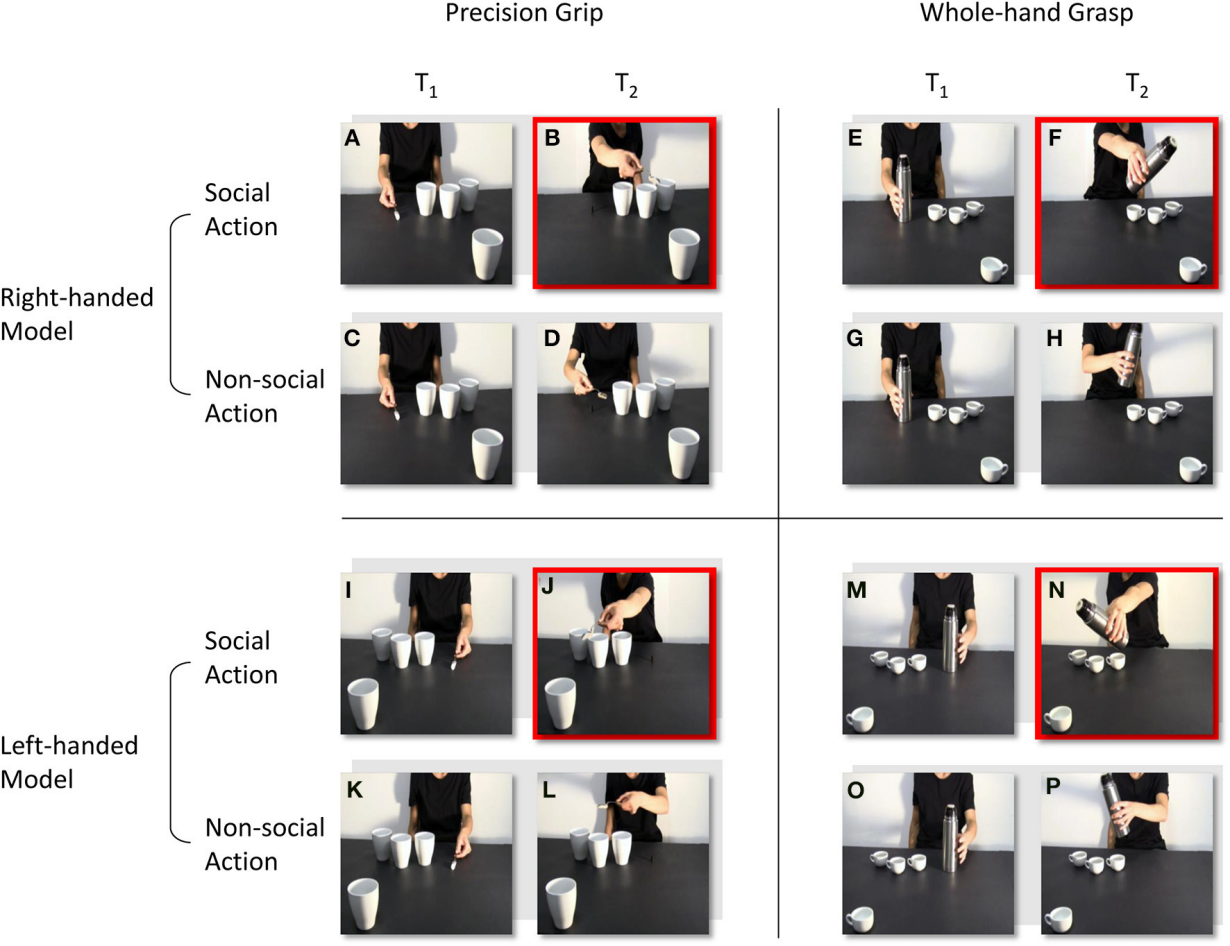
**Frames extracted from the video-clips at the time-points at which TMS pulses were delivered (T_1_ and T_2_)**. A right-handed actor reaches and grasp a sugar spoon **(A)**, then she stretches out her arm trying to pour some sugar on a cup located out of her reach **(B)**. The actor reaches and grasp the same sugar spoon **(C)**, but then she takes it back to the starting position **(D)**. The actor reaches and grasp a thermos **(E)**, then she stretches out her arm trying to pour some coffee on a coffee cup located out of her reach **(F)**. The actor reaches and grasp the same thermos **(G)**, but then she takes it back to the starting position **(H)**. In **(I–P)** video clips are reflected on a horizontal plane so that the actor appears to perform the same social and non-social actions, but with her left-hand. T_1_ and T_2_ are time-locked at the moment the actor makes contact with the object, and at the end of the action sequence. Red squares highlight the frames in which the out-of-reach object located in the video foreground elicits a complementary reaction: either a WHG **(B, J)** or a PG **(F, N)**.

The stimuli were four digitally recorded video clips showing a right-handed actor naturally reaching and grasping an object located close to her hand (Figures 1A–H): in the first, the actor reached and grasped a sugar spoon (a), poured some sugar on three cups located nearby and then stretched out her arm trying to pour some sugar on a forth cup located out of her reach (b); in the second, the actor reached and grasped a sugar spoon (c), poured some sugar on three cups located nearby and then took back the sugar spoon to the starting point (d); in the third, the actor reached and grasped a thermos (e), poured some coffee on three coffee cups located nearby and then stretched out her arm trying to pour some coffee on a forth coffee cup located out of her reach (f); in the fourth and last, an actor was shown reaching and grasping a thermos (g), pouring some coffee on three coffee cups located nearby and then taking back the thermos to the starting point (h). The four video clips were then reflected on a horizontal plane using video editing procedures so that the actor appeared to be reaching and grasping the same object with her left hand (Figures 1 I–P), for a total of eight video clips. All of the videos were taken from a frontal view, clearly showing the model grasping the sugar spoon with a precision grip (PG; i.e., the opposition of the thumb with the index finger) and the thermos with a whole-hand grasp (WHG; i.e., the opposition of the thumb with the other fingers). Crucially, the out-of-reach object was located in the video foreground, closer to the participant watching the video, thus eliciting a complementary reaction with a whole-hand grasp on the big cup and with a precision grip on the coffee cup respectively. A preliminary pilot investigation, carried out with a questionnaire and the assistance of a group of 10 participants with characteristics that were similar to those participating in the study experiment, confirmed that the social type of action (i.e., the actor leaning toward the observer) was recognized by the participants as a request to grasp the salient object (98% of positive responses).

### Data recording

#### Transcranial magnetic stimulation

Single-pulse TMS (pulse characteristics: 100 μs rise time, 1 ms duration) was delivered using a 70 mm figure-of-eight coil (Magstim polyurethane-coated coil) connected to a Magstim BiStim^2^ stimulator (The Magstim Company, UK). Pulses were delivered to the left and right M1 areas corresponding to the hand region in two separate blocks (“left M1” and “right M1” blocks, respectively). The coil was placed tangentially on the scalp, with the handle pointing laterally and caudally (Brasil-Neto et al., 1992; Mills et al., 1992). The coil was positioned in correspondence with the optimal scalp position (OSP), defined as the position at which TMS pulses of slightly suprathreshold intensity consistently produced the largest MEP from the ADM muscle. The OSP was determined by moving the intersection of the coil in approximately 0.5 cm steps around the target area until a position was reached at which a maximal MEP amplitude was produced in the target muscle with a minimal stimulation intensity. This position was marked on a tight-fitting cap that each participant was asked to wear. During the experimental sessions the coil was held by a tripod with an articulated arm. The position and orientation of the coil over the OSP was recorded and loaded into the Brainsight 2.0 neuronavigation system (Rogue Research, Montreal QC) to maintain accurate placement of the coil throughout the experiment. Defined as the minimum stimulation intensity on the OSP that induced reliable MEPs (≥50 μV peak-to-peak amplitude) in a relaxed muscle of the dominant hand in five out of ten consecutive trials, the individual resting motor threshold (rMT) was determined for each participant (Rossini et al., 1994). The same stimulation intensity (110% of the rMT) was used for the left and right M1 sessions in each subject. Stimulation intensity during the recording session ranged between 40 and 70% of the maximum stimulator output intensity (mean 53%) for the right-handed participants. For the left-handed participants, it ranged between 39 and 61% of the maximum stimulator output intensity (mean 54%).

#### Electromyography

MEPs were recorded from the first dorsal interosseus (FDI) and abductor digiti minimi (ADM) muscles of the right and left arms in separate blocks. Electromyography (EMG) activity was recorded through pairs of surface Ag-AgCl cup electrodes (9 mm diameter) placed in a belly-tendon montage. The ground electrode was placed over the participants' ipsilateral wrist. Electrodes were connected to an isolable portable ExG input box linked to the main EMG amplifier for signal transmission via a twin fiber optic cable (Professional BrainAmp ExG MR). The raw myographic signals were band-pass filtered (20 Hz–1 kHz), amplified prior to being digitalized (5 KHz sampling rate), and stored on a computer for off-line analysis. EMG data were recorded for a 300 ms interval. The interval was time-locked to the delivery of each magnetic stimulation pulse and began 100 ms prior to the onset of stimulation and ended 200 ms post-stimulation. Trials in which any EMG activity was present in the time window preceding the TMS pulse were discarded to prevent contamination of MEP measurements by background EMG activity.

### Procedure

**Figure 2:**
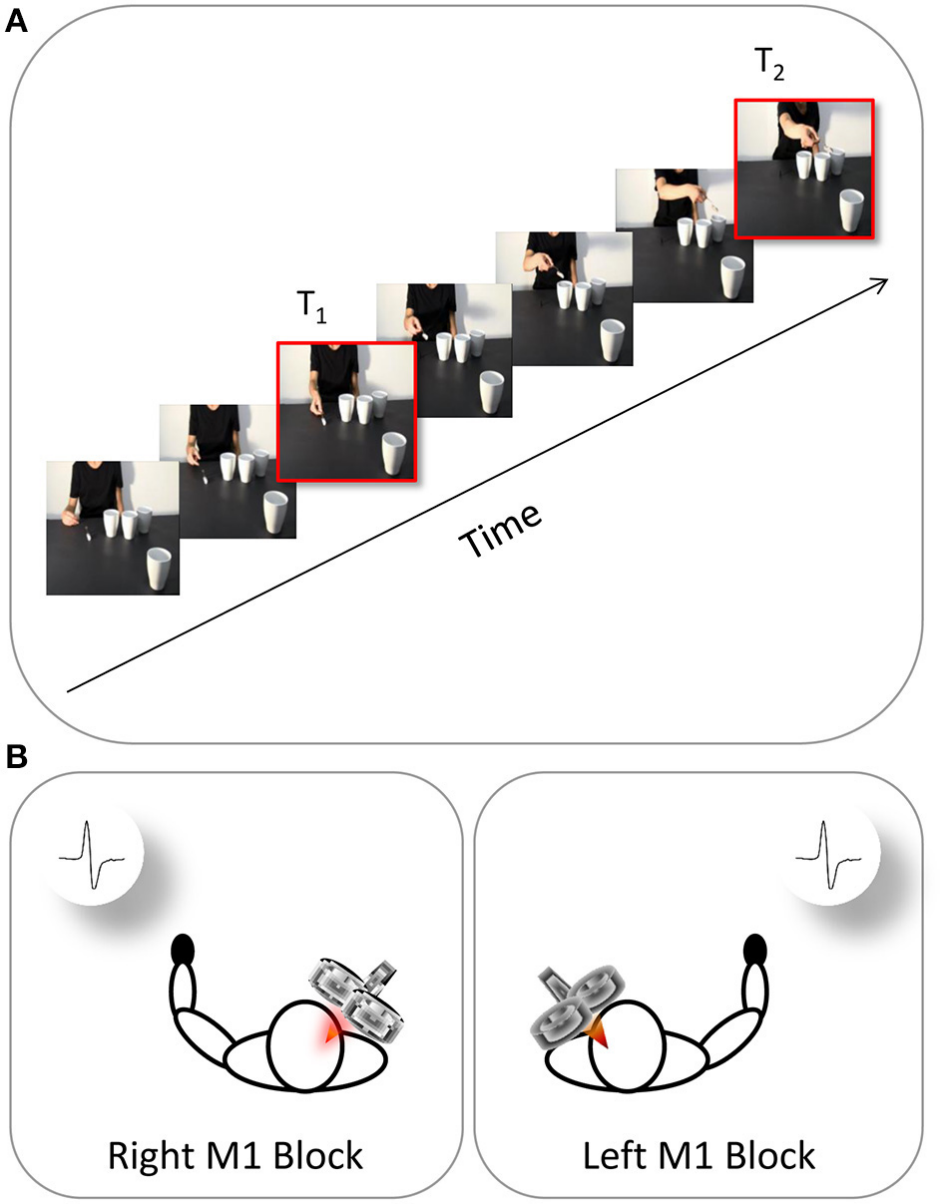
**Time and location of TMS stimulation**. The continuous oblique line represents the duration of video-clip presentation. **(A)** During each video presentation (e.g., a social action performed by the right-handed actor), TMS was delivered at two different time points (T_1_, T_2_). **(B)** EMG recordings were collected at these time points from both the participant's left hand (right M1 block) and right hand (left M1 block).

The participants were tested individually in a sound-attenuated Faraday room during a single experimental session lasting approximately 40 min and consisting in two blocks (left M1, right M1). Each participant was directed to sit in a comfortable armchair with his/her head positioned on a fixed head rest so that the eye–screen distance was 80 cm. Both arms were positioned on full-arm supports. Each participant was instructed to keep his/her hands in a prone position and as still and relaxed as possible. The task was to pay attention to the visual stimuli presented on a 19” monitor (resolution 1280 × 1024 pixels, refresh frequency 75 Hz, background luminance of 0.5 cd/m2) set at eye level. The participants were instructed to passively watch the video-clips and to avoid making any movements. To ensure that the participants paid attention to the contents of the video clips, they were told that they would be questioned at the end of the session about the visual stimuli presented. Electromyography recordings were made in the contralateral hand (Figure 2B). During the “left M1” blocks, TMS-induced MEPs were acquired from the participant's right ADM and FDI muscles during stimulation of the left M1. During the “right M1” blocks, MEPs were acquired from the participant's left ADM and FDI muscles during stimulation of the right M1. The order in which the two blocks were delivered was counterbalanced across participants. Prior to the video presentation, a baseline corticospinal excitability was assessed by acquiring 10 MEPs per block while the participants passively watched a white fixation cross on the black background on the computer screen. Ten more MEPs were recorded at the end of each block. By comparing the MEP amplitudes for the two baseline series it was possible to check for any corticospinal excitability changes related to TMS *per se* in each block. The average amplitude of the two collapsed series was utilized to set each participant's individual baseline for the data normalization process.

All the participants watched four types of video-clips presented in random order:

1. Social, PG: an actor (right/left handed) performs a precision grip to grasp a sugar spoon, pour some sugar and then stretching out her arm toward the observer (Figures 1A,B,I,J).
2. Non-social, PG: the same actor (right/left handed) performs the same action of pouring sugar, but then she goes back to the starting position (Figures 1C,D,K,L).
3. Social, WHG: an actor (right/left handed) performs a whole hand grasp to grip a thermos, pour some coffee and then stretching out her arm toward the observer (Figures 1E,F,M,N).
4. Non-social, WHG: the same actor (right/left handed) performs the same action of pouring coffee, but then she goes back to the starting position (Figures 1G,H,O,P).

The MEPs were recorded from the ADM muscle (i.e., the muscle serving little finger abduction) and FDI muscle (i.e., the muscle serving index finger abduction) due to their involvement respectively in WHG and PG. Crucially, each video clip was characterized by a mismatch between the type of grasp being observed (i.e., WHG) and the grip implicitly being requested to the observer (i.e., PG). Specifically, observing the grasp on the thermos and the large cup should elicit a pronounced activation in both FDI and ADM muscles because such muscles are involved in a WHG. When observing the grip on the sugar spoon and the coffee cup, instead, only MEPs recorded from the FDI muscle should reveal an increase because a PG does not imply the recruitment of the ADM muscle. A single TMS pulse was released during each video presentation at two specific time points: (i) during the frame showing the actor's fingers making contact with the object (T_1_; 1125 ms) and (ii) during the frame showing the lowest peak of the actor's arm trajectory (T_2_; 5900 ms; Figure 2A). The same timing was applied to all of the non-social conditions. The first time point (T_1_) was chosen to evaluate the motor resonant response. As recently demonstrated by Lago and Fernandez-del-Olmo (2011), a muscle-specific motor program is activated via the action observation system when the contact between an effector and an object is shown. The second time point (T_2_) was set at the lowest peak of the arm's trajectory to maximize the reaction to the implicit request, as identified by kinematics (Sartori et al., 2013c,d) and modeling studies (Chinellato et al., 2013) with stimuli similar to those adopted in the present study. The order of the videos and of the two different TMS delays were randomized within each of the two blocks. A total of 640 MEPs (4 muscles × 2 types of action × 2 actors × 2 types of grasp × 2 time points × 10 repetitions) was recorded for each participant. Prior to presenting the videos, each participant's baseline CS excitability was assessed by acquiring 10 MEPs per block while they passively watched a white-colored fixation cross on a black background on the computer screen. Ten more MEPs were recorded at the end of each block. By comparing MEP amplitudes recorded during the two baseline series it was possible to check for any CSE changes related to TMS *per se* in each block. The average amplitude of the two series was then utilized to set each participant's individual baseline for data normalization procedure. An inter-pulse interval lasting 10 s was presented between trials in order to minimize the potential risk of carryover effect of a TMS pulse on the subsequent one. During the first 5 s of the rest period, a message reminding the participants to keep their hands still and fully relaxed appeared on the screen. A fixation cross (10 × 10 mm) was presented for the remaining 5 s. Stimuli presentation, EMG recordings and timing of TMS stimulation were managed by E-Prime V2.0 software (Psychology Software Tools) running on a PC.

### Data analysis

The CS facilitation of FDI and ADM muscles was quantified at each stimulation point during each experimental condition by the MEP peak-to-peak amplitude (mV). Those amplitudes deviating more than 3 standard deviations from the mean and the trials contaminated by muscular pre-activation were excluded as outliers (<5%). A paired-sample *t*-test (2-tailed) was used to compare the amplitude of MEPs recorded during the two baseline trials carried out at the beginning and at the end of each block. Ratios were computed using the participants' individual mean MEP amplitude recorded during the two fixation-cross periods as baseline (MEP ratio = MEPobtained/MEPbaseline). We entered the MEP ratios in a mixed-design analysis of variance (ANOVA) with “muscle” (right FDI, right ADM, left FDI, left ADM), “type of action” (social, non-social), “actor” (right-handed, left-handed), “type of grasp” (PG, WHG) and “stimulation time” (T_1_, T_2_) as within-subjects factors, and “group” (right-handed, left-handed) as between-subjects factor. The sphericity of the data was verified prior to performing statistical analysis (Mauchly's test, *p* > 0.05). *Post-hoc* pairwise comparisons were carried out using *t*-tests and Bonferroni correction was applied to control *P*-values for multiple comparisons. A significance threshold of *P* < 0.05 was set for all statistical analyses.

## Results

The mean raw MEP amplitudes recorded during the two baseline series at the beginning and the end of each block were not significantly different in the right-handed participants neither during the “left M1” block [1406.15 vs. 1330.67 μV, respectively; *t*_(59)_ = 0.48, *p* = 0.63] nor the “right M1” block [1132.99 vs. 916.59 μV, respectively; *t*_(59)_ = 1.96, *p* = 0.07]. Similarly, the two baseline series were not significantly different in the left-handed participants neither during the “left M1” block [1796.58 vs. 1745.20 μV, respectively; *t*_(59)_ = 0.31, *p* = 0.76] nor the “right M1” block [1388.17 vs. 1101.22 μV, respectively; *t*_(59)_ = 1.94, *p* = 0.06]. Altogether these findings suggest that TMS *per se* did not induce any changes in corticospinal excitability during our experimental procedure. The mean MEP ratios from the left and right ADM and FDI muscles for each group are outlined in Table 1. The mixed-design ANOVA on the normalized MEP amplitudes showed a significant main effect of muscle [*F*_(3, 174)_ = 2.80, *p* < 0.05, η^2^_p_ = 0.05] and stimulation time [*F*_(1,58)_ = 22.56, *p* < 0.001, η^2^_p_ = 0.28]. The following interactions were also significant: “muscle by stimulation time” [*F*_(1, 174)_ = 3.72, *p* < 0.05, η^2^_p_ = 0.06], “muscle by actor by type of action” [*F*_(3, 174)_ = 2.98, *p* < 0.05, η^2^_p_ = 0.05], “muscle by actor by type of grasp by type of action” [*F*_(3, 174)_ = 2.74, *p* < 0.05, η^2^_p_ = 0.05], “muscle by type of grasp by type of action by stimulation time” [*F*_(3, 174)_ = 4.20, *p* < 0.05, η^2^_p_ = 0.07], “actor by type of grasp by stimulation time by group” [*F*_(1, 58)_ = 4.27, *p* < 0.05, η^2^_p_ = 0.07], “muscle by actor by type of action by group” [*F*_(3, 174)_ = 2.84, *p* < 0.05, η^2^_p_ = 0.05] and “muscle by actor by type of grasp by type of action by stimulation time” [*F*_(3, 174)_ = 4.81, *p* < 0.05, η^2^_p_ = 0.08]. The results obtained for *post-hoc* contrasts stemming from the five-way interaction are reported as follows.

**Table 1:** **Normalized mean (± s.e.m.) peak to peak amplitude of MEPs recorded from the ADM and the FDI muscles of both groups during the two stimulation blocks for each type of observed actor, observed grasp and type of action at each stimulation time point**.

**Actor's handedness**	**Type of grasp**	**Type of action**	**Stimulation time**	**Muscle**	**Stimulation site**			
					**Left M1**		**Right M1**	
					**Left-handers**	**Right-handers**	**Left-handers**	**Right-handers**
Right	PG	Social	1	ADM	1.074 (±0.069)	1.134 (±0.093)	1.178 (±0.131)	1.132 (±0.128)
Right	PG	Social	1	FDI	1.123 (±0.068)	1.146 (±0.070)	1.033 (±0.079)	1.109 (±0.095)
Right	PG	Social	2	ADM	1.157 (±0.117)	1.459 (±0.162)	1.296 (±0.198)	1.288 (±0.174)
Right	PG	social	2	FDI	1.176 (±0.079)	1.113 (±0.098)	1.065 (±0.103)	1.147 (±0.105)
Right	PG	Non-social	1	ADM	1.149 (±0.123)	1.069 (±0.091)	1.258 (±0.228)	1.173 (±0.161)
Right	PG	Non-social	1	FDI	1.088 (±0.064)	1.030 (±0.070)	1.017 (±0.096)	1.037 (±0.080)
Right	PG	Non-social	2	ADM	1.141 (±0.090)	1.122 (±0.092)	1.417 (±0.279)	1.341 (±0.192)
Right	PG	Non-social	2	FDI	1.201 (±0.083)	1.135 (±0.084)	1.158 (±0.085)	1.304 (±0.166)
Right	WHG	Social	1	ADM	1.111 (±0.082)	1.316 (±0.141)	1.453 (±0.286)	1.320 (±0.280)
Right	WHG	Social	1	FDI	1.078 (±0.064)	1.020 (±0.074)	0.846 (±0.061)	0.873 (±0.071)
Right	WHG	Social	2	ADM	1.131 (±0.093)	1.129 (±0.080)	1.586 (±0.337)	1.409 (±0.212)
Right	WHG	social	2	FDI	1.167 (±0.066)	1.168 (±0.110)	1.119 (±0.090)	1.185 (±0.117)
Right	WHG	Non-social	1	ADM	1.068 (±0.070)	1.175 (±0.110)	1.320 (±0.176)	1.368 (±0.257)
Right	WHG	Non-social	1	FDI	1.128 (±0.073)	1.039 (±0.085)	1.053 (±0.085)	1.057 (±0.098)
Right	WHG	Non-social	2	ADM	1.164 (±0.088)	1.323 (±0.110)	1.199 (±0.163)	1.243 (±0.138)
Right	WHG	Non-social	2	FDI	1.201 (±0.072)	1.057 (±0.084)	1.117 (±0.087)	1.078 (±0.135)
Left	PG	Social	1	ADM	1.033 (±0.088)	1.208 (±0.112)	1.128 (±0.111)	1.428 (±0.233)
Left	PG	Social	1	FDI	1.129 (±0.065)	1.213 (±0.093)	0.995 (±0.080)	1.155 (±0.113)
Left	PG	Social	2	ADM	1.063 (±0.058)	1.119 (±0.100)	1.684 (±0.316)	1.568 (±0.357)
Left	PG	Social	2	FDI	1.217 (±0.077)	1.047 (±0.116)	1.068 (±0.087)	1.074 (±0.106)
Left	PG	Non-social	1	ADM	1.112 (±0.091)	1.227 (±0.104)	1.313 (±0.194)	1.414 (±0.318)
Left	PG	Non-social	1	FDI	1.106 (±0.055)	1.183 (±0.096)	1.029 (±0.071)	1.040 (±0.080)
Left	PG	Non-social	2	ADM	1.118 (±0.085)	1.178 (±0.094)	1.283 (±0.155)	1.510 (±0.310)
Left	PG	Non-social	2	FDI	1.218 (±0.078)	1.211 (±0.096)	1.089 (±0.096)	1.154 (±0.111)
Left	WHG	Social	1	ADM	1.127 (±0.110)	1.113 (±0.088)	1.171 (±0.153)	1.339 (±0.213)
Left	WHG	Social	1	FDI	1.070 (±0.072)	1.063 (±0.082)	0.925 (±0.067)	1.073 (±0.079)
Left	WHG	Social	2	ADM	1.127 (±0.088)	1.085 (±0.118)	1.254 (±0.203)	1.299 (±0.175)
Left	WHG	Social	2	FDI	1.236 (±0.082)	1.096 (±0.115)	1.064 (±0.101)	1.238 (±0.129)
Left	WHG	Non-social	1	ADM	1.087 (±0.065)	1.104 (±0.084)	1.460 (±0.297)	1.152 (±0.139)
Left	WHG	Non-social	1	FDI	1.105 (±0.068)	1.063 (±0.095)	1.062 (±0.082)	0.913 (±0.070)
Left	WHG	Non-social	2	ADM	1.132 (±0.083)	1.143 (±0.096)	1.655 (±0.245)	1.605 (±0.303)
Left	WHG	Non-social	2	FDI	1.160 (±0.066)	1.109 (±0.095)	1.059 (±0.090)	1.141 (±0.086)

### Effects of motor resonance

#### Left-handed actor

*Post-hoc* comparisons revealed a reliable activation in all of the participants' left hand when observing a left-handed actor. In particular, observing the left-handed actor grasping a thermos at T_1_ with both a social and non-social type of action induced a greater activation in the left ADM muscle compared to the ipsilateral FDI muscle (*p*_*s*_ < 0.05; Table 1). This was confirmed for the non-social type of action at T_2_ by an increase in the left ADM muscle compared to the ipsilateral FDI muscle (*p* < 0.05; Table 1) and compared to the video in which the actor was grasping a sugar spoon (non-social type of action; *p* < 0.05; Table 1). Interestingly, observing the left-handed actor holding a thermos in the non-social type of action at T_2_ prompted a greater activation in the left ADM muscle that observing the very same action performed by the right-handed actor (*p* < 0.05; Table 1). Furthermore, *post-hoc* analysis on the four-way interaction “actor by muscle by type of action by group” showed that observing the left-handed actor performing a non-social action induced a greater activation in the left hand of both right and left-handers, with respect to their ipsilateral FDI muscles (*p*_*s*_ < 0.05). This suggests that motor resonance to an observed action performed by a left-handed actor is likely to activate the anatomically corresponding muscles (i.e., left hand) in both right- and left-handers.

#### Right-handed actor

*Post-hoc* comparisons revealed a mixed pattern of activation when observing the right-handed actor. In particular, a classical increase in the right ADM muscle was found when observing the actor performing a WHG on the thermos compared to a PG on the sugar spoon for both the social and non-social types of actions at T_1_ and T_2_ (*p*_*s*_ < 0.05; Table 1). But observing the right-handed actor grasping a thermos (WHG) at T_1_ also induced an increase in both right and left ADM muscles with respect to the corresponding ipsilateral FDI muscles (*p*_*s*_ <0.05; Table 1). This seems to suggest that participants were resonating with both hands. Statistically significant differences were also found in both FDI muscles when observing a WHG compared to a PG at T_1_ and T_2_ (*p*_*s*_ < 0.05; Table 1). These results are in line with the literature on reach-to-grasp kinematics, suggesting a major involvement of FDI during precision grips compared to whole-hand grasps (Sartori et al., 2012).

### Effects of reciprocity

#### Left-handed actor

**Figure 3:**
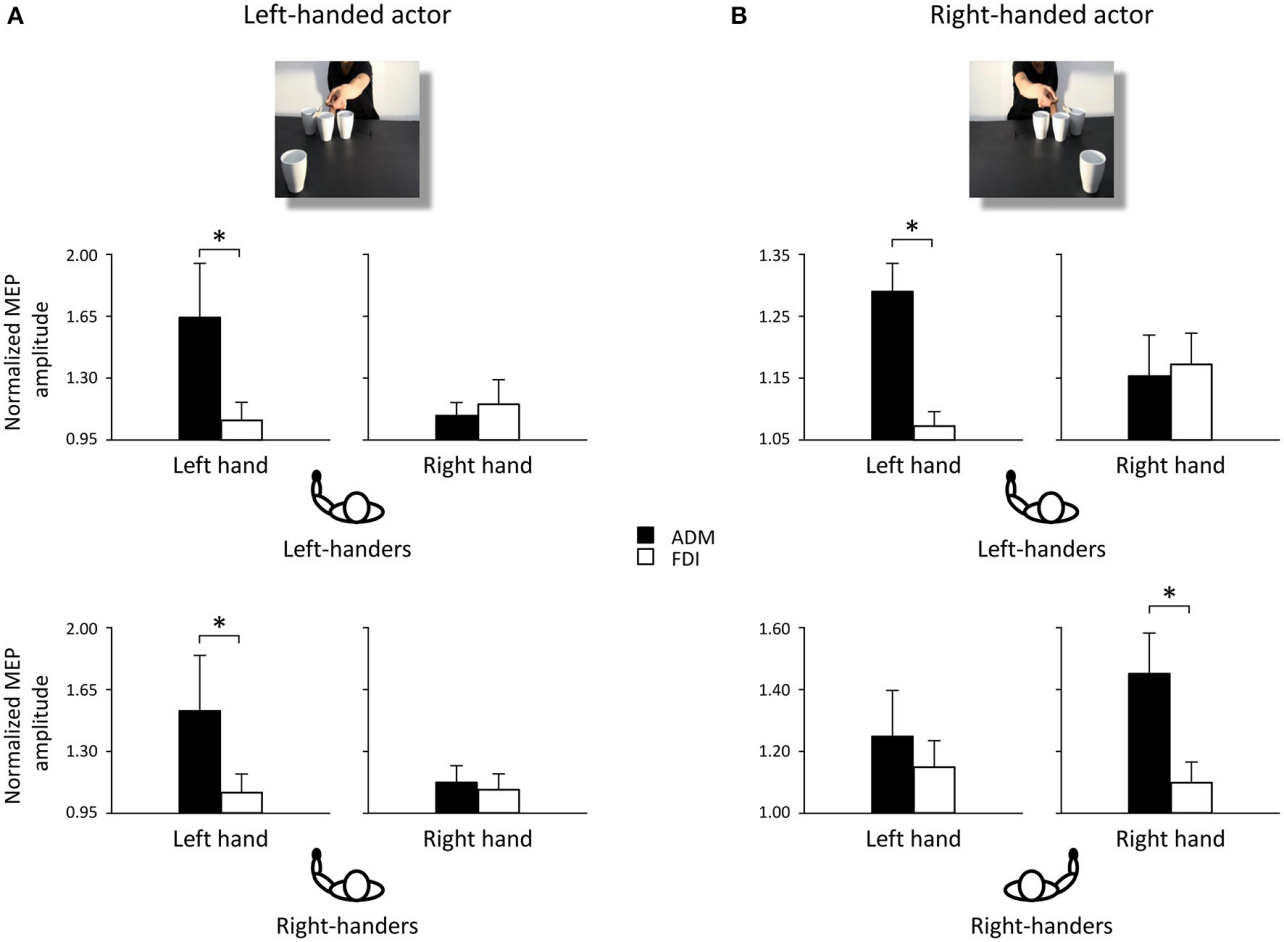
**Normalized mean MEP amplitude for ADM (black bars) and FDI (white bars) muscles when observing a left-handed (A) and a right-handed actor (B) performing a social PG**. Asterisks indicate significant comparisons (*p* < 0.05). Bars represent the standard error of means. Black hands of schematic drawings representing the participants highlight that left-handers activate the left hands independently from the object's location. Whereas right-handers activate the left hand when the object is located to their left side, and the right hand when the object is located to their right side.

*Post-hoc* comparisons revealed that observing the left-handed actor holding the sugar spoon and leaning toward the out of reach cup eliciting a WHG in the participant's hand induced a predictable increase in the left ADM muscle at T_2_ with respect to T_1_ (p < 0.05). The same was found with respect to the contralateral ADM muscle (*p* < 0.05), to the ipsilateral FDI muscle (*p* < 0.05), to the non-social type of action showing the actor simply holding the sugar spoon back to the starting point (*p* < 0.05), to the other social action eliciting a PG toward the coffee cup (*p* < 0.05), and to the very same action performed by the right-handed actor. A significant decrease in MEPs activity was also found in the left ADM muscle when observing the actor holding the thermos and leaning toward the out of reach coffee cup eliciting a PG in the participant's hand, with respect to the non-social action (*p* < 0.05). Observing the left-handed actor performing a complementary request in the social types of actions induced in right-handers a greater muscular activation of left hand muscles with respect to observing the non-social actions (*p*_*s*_ < 0.05). Interestingly, a greater activation of the left ADM muscle was found in right-handers and left-handers with respect to their ipsilateral FDI muscles (*p*_*s*_ < 0.05; Figure 3A) for the social PG actions performed by the left-handed actor.

#### Right-handed actor

*Post-hoc* comparisons revealed that observing the right-handed actor holding the sugar spoon and leaning toward the out of reach cup eliciting a WHG in the participant's hand induced a predictable increase in the right ADM muscle at T_2_ with respect to T_1_ (*p* < 0.05). The same was found with respect to the ipsilateral FDI muscle (*p* < 0.05), to the non-social type of action showing the actor simply holding the sugar spoon back to the starting point (*p* < 0.05), to the other social action eliciting a PG toward the coffee cup (*p* < 0.05), and to the very same action performed by the left-handed actor. A significant decrease in activation was also found in the right ADM muscle when observing the other video clip showing the actor holding the thermos and leaning toward the out of reach coffee cup eliciting a PG in the participant's hand, with respect to the non-social action of holding the thermos back to the starting point (*p* < 0.05). The effect of complementary activation previously described in the literature was confirmed (Sartori et al., 2012, 2013b,c,d). But an increase was found at T_2_ also in the left ADM muscle with respect to the ipsilateral FDI muscle (*p* < 0.05) for the social type of action requiring a WHG on the cup. Interestingly, observing the right-handed actor performing a complementary request induced a greater muscular activation with respect to observing the non-social actions (*p*_*s*_ < 0.05; Table 1). An effect supported by previous literature (Sartori et al., 2011). But this increase was only evident in the right hand of right-handers and in the left hand of left-handers, suggesting that they translated the observed movement into their dominant effector for planning the most appropriate response. This was confirmed by the greater activation of the right ADM muscle in right-handers and of the left ADM muscle in left-handers with respect to their ipsilateral FDI muscles (*p*_*s*_ < 0.05; Figure 3B) for the social PG actions performed by the right-handed actor.

## Discussion

The main aim of the present study was to bring a substantial advancement in our knowledge of the role played by hand dominance in modulating motor resonant and complementary responses in social contexts. Are motor resonance and reciprocity shaped by handedness?

To test this issue, we adopted video clips showing a right-handed actor performing social and non-social actions eliciting in the observer congruent and incongruent types of motor activations, along with the very same actions performed by a left-handed actor (i.e., obtained through digital flipping of the original ones). Participants were both right and left-handers. Results show that, independently from group handedness, motor resonance effects strictly linked to the observed muscles emerged in all participants, though with a more unilateral pattern of activations when observing a left- with respect to a right-handed actor. This effect could be explained on the basis of previous findings showing that left-handers tend to translate any observed motor program into their dominant effector (Sartori et al., 2013a). This is in agreement with previous evidence of more bilaterally spread brain functions in left-than in right-handers (Matsuo et al., 2002; Jorgens et al., 2007; Krombholz, 2008; Müller et al., 2011). In neural terms, very few studies have tried to shed light on the underpinnings of hand grasping actions in both right-and left-handers (e.g., Begliomini et al., 2008). In this respect, evidence suggests a specific right hemisphere contribution to grip formation (Hermsdorfer et al., 1999; Farne et al., 2003), and in particular a significant role of the right dorsal premotor cortex (dPMC) in the control of goal-related hand movements depending on handedness (Begliomini et al., 2008). Specifically, a similar activity within the right dPMC for both right-and left-handers was found when they performed the task with the right hand, and a different activity between the two groups was found when the left hand was used. This was evident when looking at the significant increase in activation when left-handers used the dominant left rather than the right hand. This observation is in line with our data demonstrating a preferential leftward hand activation in left-handers observing both left-and right-handers, and with the anatomical observation of differences in inter-hemispheric connections in relation to handedness (Amunts et al., 2000). And it might also suggest differences in the functional organization motor and premotor areas in right- and left-handed people (Solodkin et al., 2001).

In view of the fact that motor resonance reflects the motor representation evoked by a perceived action in an observer, our results suggest that in the context of a social request directed to the observers, independently from their handedness, the perceptual-motor matching of the observed action give the way to an incongruent activation in the muscles directly involved in the interaction. That is, motor activation in right handers is found in the right hand when the actor asks for a right complementary gesture and in the left hand when the actor asks for a left complementary action. This supports the hypothesis of a sophisticated model of motor resonance. The direct-matching hypothesis postulates that viewing an action automatically evokes in the observer a representation of the motor commands necessary to execute that same action. TMS experiments typically show that observed movements are processed in a strictly time-locked, muscle specific fashion (Baldissera et al., 2001; Gangitano et al., 2001; Borroni et al., 2005; Montagna et al., 2005; Borroni and Baldissera, 2008; Candidi et al., 2008; Alaerts et al., 2009; Cavallo et al., 2011). However, when a complementary reaction is implicitly required by the observed agent, incongruent patterns of motor activations take place (Sartori et al., 2012, 2013b,c,d; Hamilton, 2013). The findings outlined here, suggesting that the perceptual-motor mapping of a movement is also sensitive to the observed handedness complement those studies and take research one step further.

Another explanation for this effect could be ascribed to the motor coding of action affordance elicited by the salient object in the social type of action. This would point to a mechanism for recognizing “social affordances,” that is specific types of affordances (Gibson, 1979; Jeannerod, 1994; Craighero et al., 1998; Tucker and Ellis, 1998; Buccino et al., 2009) produced by the establishment of a shared intentional space (Tomasello, 1999). The implicit request by the actor -facing the participants-toward the object inside their peripersonal space is a crucial aspect which favors a readiness to engage in a complementary interaction (Costantini et al., 2010, 2011). In line with this, we specifically devised control conditions in which the actor was finally directed to bring her hand back to its initial position, despite the presence of the fourth object still visible in the foreground. That control conditions were created in order to detach the role of the intentional request from object affordances. Indeed, the present results seem to suggest that only making affordances salient evokes a readiness to enact them. As long as an object becomes relevant to the goal of an action, it is conceivable that a highly efficient mechanism enables subjects to correctly plan movements toward this target in a functional action-specific mode. And this indeed happens in right-handers. Whereas left-handers tend to persistently activate their dominant effector. With respect to the relation between motor resonance, reciprocity, and dominance, our results extend previous evidence, showing that the observed handedness differently shapes motor resonant and complementary reactions in right-and left-handers. Assuming that this modulation might be an index of motor representations' capability of taking into account the observed hand dominance and the target location, the findings outlined here can support the evidence of a sophisticated mechanism allowing right handers to plan movements toward the target in a functional action-specific mode and left-handers to convert another person's pattern of movement into their optimal motor commands.

## Author contributions

Luisa Sartori contributed to the acquisition, analysis and interpretation of data, and to drafting the work. Chiara Begliomini contributed revising the draft critically. Giulia Panozzo and Alice Garolla contributed to the acquisition of data for the work. Umberto Castiello contributed to drafting the work and revising it for important intellectual content.

### Conflict of interest statement

The authors declare that the research was conducted in the absence of any commercial or financial relationships that could be construed as a potential conflict of interest.
